# Aging x Environment x genetic risk for late onset Alzheimer’s disease results in alterations in cognitive function in mice independent of amyloid and tau pathology

**DOI:** 10.1002/alz.093301

**Published:** 2025-01-03

**Authors:** Sean‐Paul Gerard Williams, Diogo Francisco Silva Santos, Kathryn A Haynes, Nicholas Heaton, Jason T Hart, Kevin P Kotredes, Ravi S Pandey, Scott C Persohn, Kierra Eldridge, Cynthia M Ingraham, Christopher D Lloyd, Nian Wang, Michael Sasner, Gregory W Carter, Paul R Territo, Bruce T. Lamb, Gareth R Howell, Adrian L Oblak, Stacey J Sukoff Rizzo

**Affiliations:** ^1^ University of Pittsburgh Aging, Pittsburgh, PA USA; ^2^ University of Pittsburgh School of Medicine, PITTSBURGH, PA USA; ^3^ University of Pittsburgh School of Medicine, Pittsburgh, PA USA; ^4^ The Jackson Laboratory, Bar Harbor, ME USA; ^5^ The Jackson Laboratory for Genomic Medicine, Farmington, CT USA; ^6^ Stark Neurosciences Research Institute, Indiana University School of Medicine, Indianapolis, IN USA; ^7^ Indiana University School of Medicine, Indianapolis, IN USA; ^8^ Stark Neurosciences Research Institute, indianapolis, IN USA; ^9^ Indiana University, Indianapolis, IN USA

## Abstract

**Background:**

Alzheimer’s disease (AD) research has been historically dominated with studies in mouse models expressing familial AD mutations; however, the majority of AD patients have the sporadic, late‐onset form of AD (LOAD). To address this gap, the IU/JAX/PITT MODEL‐AD Consortium has focused on development of mouse models that recapitulate LOAD by combining genetic risk variants with environmental risk factors and aging to enable more precise models to evaluate potential therapeutics. The present studies were undertaken to characterize cognitive and neurophysiological phenotypes in LOAD mice.

**Method:**

Two genetic risk factors, *APOE4* and Trem2*R47H, were incorporated into C57BL/6J mice with humanized amyloid‐beta to produce the LOAD2 model (JAX# 030670). Male and female LOAD2 and WT mice were exposed to ad libitum 45% high‐fat diet from 2‐months of age (LOAD2+HFD or WT+HFD, respectively) throughout their lifespan and compared to LOAD2 and WT mice on control diet (+CD). Cognitive training began at 14‐months of age using a touchscreen testing battery, similar to previously described methods (Oomen et al 2013). At the conclusion of touchscreen testing, subjects were implanted with wireless telemetry devices (DSI) for evaluation of electroencephalography (EEG) signatures.

**Result:**

All subjects met the touch‐reward association criteria. During task acquisition LOAD2+CD mice demonstrated impaired acquisition relative to WT+CD, while both LOAD2+HFD and WT+HFD failed to learn the task as indicated by accuracy less than chance (<50%); which was confirmed in a separate cohort. LOAD2+HFD mice demonstrated increased spikewave events as measured by EEG, relative to LOAD2+CD. At 18‐months of age +CD mice that met acquisition criteria were evaluated in a location discrimination task with LOAD2+CD mice demonstrating modest impairments in pattern separation relative to age‐matched WT+CD.

**Conclusion:**

These data are the first reports of cognitive deficits and neurophysiological alterations in mice with environmental x genetic risk for LOAD, independent of amyloid and tau pathology. Importantly, the present findings demonstrate the sensitivity of the translational touchscreen testing battery for detecting mild cognitive impairment in LOAD mice with corresponding neurophysiologic alterations, and extend previous characterization data for the LOAD2 model and its utility for the study of the biology of LOAD.

